# Second-generation PLINK: rising to the challenge of larger and richer datasets

**DOI:** 10.1186/s13742-015-0047-8

**Published:** 2015-02-25

**Authors:** Christopher C Chang, Carson C Chow, Laurent CAM Tellier, Shashaank Vattikuti, Shaun M Purcell, James J Lee

**Affiliations:** 1 Complete Genomics, 2071 Stierlin Court, Mountain View, 94043 CA USA; 2 BGI Cognitive Genomics Lab, Building No. 11, Bei Shan Industrial Zone, Yantian District, Shenzhen, 518083 China; 3 Mathematical Biology Section, NIDDK/LBM, National Institutes of Health, Bethesda, 20892 MD USA; 4 Bioinformatics Centre, University of Copenhagen, Copenhagen, 2200 Denmark; 5 Stanley Center for Psychiatric Research, Broad Institute of MIT and Harvard, Cambridge, 02142 MA USA; 6 Division of Psychiatric Genomics, Department of Psychiatry, Icahn School of Medicine at Mount Sinai, New York, 10029 NY USA; 7 Institute for Genomics and Multiscale Biology, Icahn School of Medicine at Mount Sinai, New York, 10029 NY USA; 8 Analytic and Translational Genetics Unit, Psychiatric and Neurodevelopmental Genetics Unit, Massachusetts General Hospital, Boston, 02114 MA USA; 9 Department of Psychology, University of Minnesota Twin Cities, Minneapolis, 55455 MN USA

**Keywords:** GWAS, Population genetics, Whole-genome sequencing, High-density SNP genotyping, Computational statistics

## Abstract

**Background:**

PLINK 1 is a widely used open-source C/C++ toolset for genome-wide association studies (GWAS) and research in population genetics. However, the steady accumulation of data from imputation and whole-genome sequencing studies has exposed a strong need for faster and scalable implementations of key functions, such as logistic regression, linkage disequilibrium estimation, and genomic distance evaluation. In addition, GWAS and population-genetic data now frequently contain genotype likelihoods, phase information, and/or multiallelic variants, none of which can be represented by PLINK 1’s primary data format.

**Findings:**

To address these issues, we are developing a second-generation codebase for PLINK. The first major release from this codebase, PLINK 1.9, introduces extensive use of bit-level parallelism, $O\left (\sqrt {n}\right)$-time/constant-space Hardy-Weinberg equilibrium and Fisher’s exact tests, and many other algorithmic improvements. In combination, these changes accelerate most operations by 1-4 orders of magnitude, and allow the program to handle datasets too large to fit in RAM. We have also developed an extension to the data format which adds low-overhead support for genotype likelihoods, phase, multiallelic variants, and reference vs. alternate alleles, which is the basis of our planned second release (PLINK 2.0).

**Conclusions:**

The second-generation versions of PLINK will offer dramatic improvements in performance and compatibility. For the first time, users without access to high-end computing resources can perform several essential analyses of the feature-rich and very large genetic datasets coming into use.

**Electronic supplementary material:**

The online version of this article (doi:10.1186/s13742-015-0047-8) contains supplementary material, which is available to authorized users.

## Findings

Because of its broad functionality and efficient binary file format, PLINK is widely employed in data-processing pipelines that are established for gene-trait mapping and population-genetic studies. However, the five years since the final first-generation update (v1.07), however, have witnessed the introduction of new algorithms and analytical approaches, the growth in size of typical datasets, as well as wide deployment of multicore processors.

In response, we have developed PLINK 1.9, a comprehensive performance, scaling, and usability update. Our data indicate that its speedups frequently exceed two, and sometimes even three, orders of magnitude for several commonly used operations. PLINK 1.9’s core functional domains are unchanged from that of its predecessor—data management, summary statistics, population stratification, association analysis, identity-by-descent estimation [1] —and it is usable as a drop-in replacement in most cases, requiring no changes to existing scripts. To support easier interoperation with newer software, for example BEAGLE 4 [2], IMPUTE2 [3], GATK [4], VCFtools [5], BCFtools [6] and GCTA [7], features such as the import/export of VCF and Oxford-format files and an efficient cross-platform genomic relationship matrix (GRM) calculator have been introduced. Most pipelines currently employing PLINK 1.07 can expect to benefit from upgrading to PLINK 1.9.

A major problem remains: PLINK’s core file format can only represent unphased, biallelic data; however we are developing a second update, PLINK 2.0, to address this.

### Improvements in PLINK 1.9

#### Bit-level parallelism

Modern ×86 processors are designed to operate on data in (usually 64-bit) machine word or (≥ 128-bit) vector chunks. The PLINK 1 binary file format supports this well: the format’s packed 2-bit data elements can, with the use of bit arithmetic, easily be processed 32 or 64 at a time. However, most existing programs fail to exploit opportunities for bit-level parallelism; instead their loops painstakingly extract and operate on a single data element at a time. Replacement of these loops with bit-parallel logic is, by itself, enough to speed up numerous operations by more than one order of magnitude.

For example, when comparing two DNA segments, it is frequently useful to start by computing their Hamming distance. Formally, define two sequences {*a*_1_,*a*_2_,…,*a*_*m*_} and {*b*_1_,*b*_2_,…,*b*_*m*_} where each *a*_*i*_ and *b*_*i*_ has a value in {0,1,2,*ϕ*}, representing either the number of copies of the major allele or (*ϕ*) the absence of genotype data. Also define an intersection set *I*_*a*,*b*_:={*i*:*a*_*i*_≠*ϕ* and *b*_*i*_≠*ϕ*}. The “identity-by-state” measure computed by PLINK can then be expressed as

$$1 - \frac{\sum_{i\in I_{a,b}}|a_{i} - b_{i}|}{2|I_{a,b}|}. $$

where |*I*_*a*,*b*_| denotes the size of set *I*_*a*,*b*_, while |*a*_*i*_−*b*_*i*_| is the absolute value of *a*_*i*_ minus *b*_*i*_. The old calculation proceeded roughly as follows:

IBS0 := 0 IBS1 := 0 IBS2 := 0 For *i*∈{1,2,…,*m*}:

- If *a*_*i*_=*ϕ* or *b*_*i*_=*ϕ*, skip
- otherwise, if *a*_*i*_=*b*_*i*_, increment IBS2
- otherwise, if (*a*_*i*_=2 and *b*_*i*_=0), or (*a*_*i*_=0 and *b*_*i*_=2), increment IBS0
- otherwise, increment IBS1

Return $\frac {0\cdot \text {IBS}0 + 1\cdot \text {IBS}1 + 2\cdot \text {IBS}2}{2\cdot (\text {IBS}0 + \text {IBS}1 + \text {IBS}2)}$

We replaced this with roughly the following, based on bitwise operations on 960-marker blocks:

$$ m^{\prime} := 960\left\lceil \frac{m}{960}\right\rceil $$

Pad the ends of {*a*_*i*_} and {*b*_*i*_} with *ϕ*s, if necessary *A*_*i*_:={01_2_ if *a*_*i*_=*ϕ*,00_2_ if *a*_*i*_=0,10_2_ if *a*_*i*_=1,11_2_ if *a*_*i*_=2} *B*_*i*_:={01_2_ if *b*_*i*_=*ϕ*,00_2_ if *b*_*i*_=0,10_2_ if *b*_*i*_=1,11_2_ if *b*_*i*_=2} *C*_*i*_:={00_2_ if *a*_*i*_=*ϕ*,11_2_ otherwise} *D*_*i*_:={00_2_ if *b*_*i*_=*ϕ*,11_2_ otherwise} diff := 0 obs := 0For *i*∈{1,961,1921,…,*m*^′^−959}:

- *E*:=*A*_*i*..*i*+959_XOR*B*_*i*..*i*+959_
- *F*:=*C*_*i*..*i*+959_AND*D*_*i*..*i*+959_
- diff := diff + popcount(*E*AND*F*)
- obs := obs + popcount(*F*)

Return $\frac {\text {obs} - \text {diff}}{\text {obs}}$.

The idea is that ({*C*_*i*_}AND {*D*_*i*_}) yields a bit vector with two ones for every marker where genotype data is present for both samples, and two 0 s elsewhere, so 2|*I*_*a*,*b*_| is equal to the number of ones in that bit vector; while (({*A*_*i*_}XOR {*B*_*i*_})AND {*C*_*i*_}AND {*D*_*i*_}) yields a bit vector with a 1 for every nucleotide difference. Refer to Additional file 1 [8] for more computational details. Our timing data (see “Performance comparisons” below) indicate that this algorithm takes less than twice as long to handle a 960-marker block as PLINK 1.07 takes to handle a single marker.

#### Bit population count

The “popcount” function above, defined as the number of ones in a bit vector, merits further discussion. Post-2008 x86 processors support a specialized instruction that directly evaluates this quantity. However, thanks to 50 years of work on the problem, algorithms exist which evaluate bit population count nearly as quickly as the hardware instruction while sticking to universally available operations. Since PLINK is still used on some older machines, we took one such algorithm (previously discussed and refined by [9]), and developed an improved SSE2-based implementation. (Note that SSE2 vector instructions are supported by even the oldest x86-64 processors).

The applications of bit population count extend further than might be obvious at first glance. As another example, consider computation of the correlation coefficient *r* between a pair of genetic variants, where some data may be missing. Formally, let *n* be the number of samples in the dataset, and {*x*_1_,*x*_2_,…,*x*_*n*_} and {*y*_1_,*y*_2_,…,*y*_*n*_} contain genotype data for the two variants, where each *x*_*i*_ and *y*_*i*_ has a value in {0,1,2,*ϕ*}. In addition, define

$${\fontsize{9}{6}\begin{aligned} I_{x,y} & := \{i: x_{i}\ne \phi\ \text{and}\ y_{i}\ne \phi \}, \\ v_{i} & := \{0\mathrm{ if }x_{i}=\phi, (x_{i}-1)\text{otherwise}\}, \\ w_{i} & := \{0\mathrm{ if }y_{i}=\phi, (y_{i}-1)\text{otherwise}\}, \\ \overline{v} & := |I_{x,y}|^{-1}\sum_{i\in I_{x,y}}v_{i}, \\ \overline{w} & := |I_{x,y}|^{-1}\sum_{i\in I_{x,y}}w_{i}, \\ \end{aligned}} $$

$${\fontsize{9}{6}\begin{aligned} \overline{v^{2}} & := |I_{x,y}|^{-1}\sum_{i\in I_{x,y}}{v_{i}^{2}},\text{and} \\ \overline{w^{2}} & := |I_{x,y}|^{-1}\sum_{i\in I_{x,y}}{w_{i}^{2}}. \end{aligned}} $$

The correlation coefficient of interest can then be expressed as

$$\begin{array}{@{}rcl@{}} r & = & \frac{|I_{x,y}|^{-1}\sum_{i\in I_{x,y}}\left(v_{i} - \overline{v}\right)\left(w_{i} - \overline{w}\right)}{\sqrt{\left(\overline{v^{2}} - \overline{v}^{2}\right)\left(\overline{w^{2}} - \overline{w}^{2}\right)}}  \\ & = & \frac{|I_{x,y}|^{-1}\sum_{i=1}^{n}v_{i}w_{i} - \overline{v}\cdot \overline{w}}{\sqrt{\left(\overline{v^{2}} - \overline{v}^{2}\right)\left(\overline{w^{2}} - \overline{w}^{2}\right)}} \end{array} $$

Given PLINK 1 binary data, |*I*_*x*,*y*_|, $\overline {v}$, $\overline {w}$, $\overline {v^{2}}$, and $\overline {w^{2}}$ can easily be expressed in terms of bit population counts. The dot product $\sum _{i=1}^{n}v_{i}w_{i}$ is trickier; to evaluate it, we preprocess the data so that the genotype bit vectors *X* and *Y* encode homozygote minor calls as 00_2_, heterozygote *and* missing calls as 01_2_, and homozygote major calls as 10_2_, and then proceed as follows:

1. Set *Z* := (*X*OR*Y*) AND01010101… _2_
2. Evaluate
  popcount2(((*X*XOR*Y*) AND (10101010… _2_ - *Z*)) OR*Z*),
  where popcount2() sums 2-bit quantities instead of counting set bits. (This is actually cheaper than PLINK’s regular population count; the first step of software popcount() is reduction to a popcount2() problem).
3. Subtract the latter quantity from *n*.

The key insight behind this implementation is that each *v*_*i*_*w*_*i*_ term is in {−1,0,1}, and can still be represented in 2 bits in an addition-friendly manner. (This is not strictly necessary for bitwise parallel processing—the partial sum lookup algorithm discussed later handles 3-bit outputs by padding the raw input data to 3 bits per genotype call—but it allows for unusually high efficiency). The exact sequence of operations that we chose to evaluate the dot-product terms in a bitwise parallel fashion is somewhat arbitrary.

We note that when computing a matrix of correlation coefficients between all pairs of variants, if no genotype data is absent, then |*I*_*x*,*y*_| is invariant, $\overline {v}$ and $\overline {v^{2}}$ do not depend on *y*, and $\overline {w}$ and $\overline {w^{2}}$ do not depend on *x*. Thus, these five values would not need to be recomputed for each variant pair at *O*(*m*^2^*n*) total time cost; they could instead be precomputed outside the main loop at a total cost of *O*(*mn*) time and *O*(*m*) space. PLINK 1.9 optimizes this common case.

See popcount_longs() in plink_common.c for our primary bit population count function, and plink_ld.c for several correlation coefficient evaluation functions.

#### Multicore and cluster parallelism

Modern x86 processors also contain increasing numbers of cores, and computational workloads in genetic studies tend to contain large “embarrassingly parallel” steps which can easily exploit additional cores. Therefore, PLINK 1.9 autodetects the number of cores present in the machine it is running on, and many of its heavy-duty operations default to employing roughly that number of threads. (This behavior can be manually controlled with the –threads flag.) Most of PLINK 1.9’s multithreaded computations use a simple set of cross-platform C functions and macros, which compile to pthread library idioms on Linux and OS X, and OS-specific idioms like _beginthreadex() on Windows.

PLINK 1.9 also contains improved support for distributed computation: the –parallel flag makes it easy to split large matrix computations across a cluster, while –write-var-ranges simplifies splitting of per-variant computations.

Graphics processing units (GPUs) remain as a major unexploited computational resource. We have made the development of GPU-specific code a low priority since their installed base is much smaller than that of multicore processors, and the speedup factor over well-written multithreaded code running on similar-cost, less specialized hardware is usually less than 10x [10,11]. However, we do plan to build out GPU support for the heaviest-duty computations after most of our other PLINK 2 development goals are achieved.

#### Memory efficiency

To make it possible for PLINK 1.9 to handle the huge datasets that benefit the most from these speed improvements, the program core no longer keeps the main genomic data matrix in memory; instead, most of its functions only load data for a single variant, or a small window of variants, at a time. Sample × sample matrix computations still normally require additional memory proportional to the square of the sample size, but –parallel gets around this:


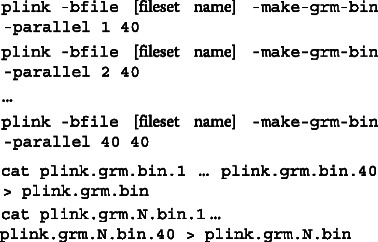


calculates 1/40th of the genomic relationship matrix per run, with correspondingly reduced memory requirements.

#### Other noteworthy algorithms

##### Partial sum lookup

Each entry of a weighted genomic distance matrix between pairs of individuals is a sum of per-marker terms. Given PLINK 1 binary data, for any specific marker, there are seven distinct cases at most:

1. Both genotypes are homozygous for the major allele.
2. One is homozygous major, and the other is heterozygous.
3. One is homozygous major, and the other is homozygous minor.
4. Both are heterozygous.
5. One is heterozygous, and the other is homozygous minor.
6. Both are homozygous minor.
7. At least one genotype is missing.

For example, the GCTA genomic relationship matrix is defined by the following per-marker increments, where *q* is the minor allele frequency:

1. $\frac {(2-2q)(2-2q)}{2q(1-q)}$
2. $\frac {(2-2q)(1-2q)}{2q(1-q)}$
3. $\frac {(2-2q)(0-2q)}{2q(1-q)}$
4. $\frac {(1-2q)(1-2q)}{2q(1-q)}$
5. $\frac {(1-2q)(0-2q)}{2q(1-q)}$
6. $\frac {(0-2q)(0-2q)}{2q(1-q)}$
7. 0; subtract 1 from the final denominator instead, in another loop

This suggests the following matrix calculation algorithm, as a first draft:

1. Initialize all distance/relationship partial sums to zero.
2. For each marker, calculate and save the seven possible increments in a lookup table, and then refer to the table when updating partial sums. This replaces several floating point adds/multiplies in the inner loop with a single addition operation.

We can substantially improve on this by handling multiple markers at a time. Since seven cases can be distinguished by three bits, we can compose a sequence of operations which maps a pair of padded 2-bit genotypes to seven different 3-bit values in the appropriate manner. On 64-bit machines, 20 3-bit values can be packed into a machine word—for example, let bits 0-2 describe the relation at marker #0, bits 3-5 describe the relation at marker #1, and so forth, all the way up to bits 57-59 describing the relation at marker #19—so this representation lets us instruct the processor to act on 20 markers simultaneously.

Then, we need to perform the update

$$A_{jk} := A_{jk} + f_{0}(x_{0}) + f_{1}(x_{1}) + \ldots + f_{19}(x_{19}) $$

where the *x*_*i*_’s are bit trios, and the *f*_*i*_’s map them to increments. This could be done with 20 table lookups and floating point addition operations. Or, the update could be restructured as

$$A_{jk} := A_{jk} + f_{\{0-4\}}(x_{\{0-4\}}) + \ldots + f_{\{15-19\}}(x_{\{15-19\}}) $$

where *x*_{0−4}_ denotes the lowest-order **15** bits, and *f*_{0−4}_ maps them directly to *f*_0_(*x*_0_)+*f*_1_(*x*_1_)+*f*_2_(*x*_2_)+*f*_3_(*x*_3_)+*f*_4_(*x*_4_); similarly for *f*_{5−9}_, *f*_{10−14}_, and *f*_{15−19}_. In exchange for some precomputation—four tables with 2^15^ entries each; total size 1 MB, which is not onerous for modern L2/L3 caches—this restructuring licenses the use of four table lookups and adds per update instead of twenty. See fill_weights_r() and incr_dists_r() in plink_calc.c for source code.

##### Hardy-Weinberg equilibrium and Fisher’s exact tests

Under some population genetic assumptions such as minimal inbreeding, genotype frequencies for a biallelic variant can be expected to follow the Hardy-Weinberg proportions

$$\begin{aligned} &\text{freq} (A_{1}A_{1}) = p^{2} \qquad \text{freq} (A_{1}A_{2}) = 2pq\qquad\\ &\text{freq} (A_{2}A_{2}) = q^{2} \end{aligned} $$

where *p* is the frequency of allele *A*_1_ and *q*=1−*p* is the frequency of allele *A*_2_ [12]. It is now common for bioinformaticians to use an exact test for deviation from Hardy-Weinberg equilibrium (HWE) to help detect genotyping error and major violations of the Hardy-Weinberg assumptions.

PLINK 1.0 used the SNP-HWE algorithm in a paper by Wigginton et al. [13] to execute this test. SNP-HWE exploits the fact that, while the absolute likelihood of a contingency table involves large factorials which are fairly expensive to evaluate, the ratios between its likelihood and that of adjacent tables are simple since the factorials almost entirely cancel out [14]. More precisely, given *n* diploid samples containing a total of *n*_1_ copies of allele *A*_1_ and *n*_2_ copies of allele *A*_2_ (so *n*_1_+*n*_2_=2*n*), there are $\frac {(2n)!}{n_{1}!n_{2}!}$ distinct ways for the alleles to be distributed among the samples, and $\frac {(2^{n_{12}})(n!)}{((n_{1}-n_{12})/2)!n_{12}!((n_{2}-n_{12})/2)!}$ of those ways correspond to exactly *n*_12_ heterozygotes when *n*_12_ has the same parity as *n*_1_ and *n*_2_. Under Hardy-Weinberg equilibrium, each of these ways is equally likely. Thus, the ratio between the likelihoods of observing exactly *n*_12_=*k*+2 heterozygotes and exactly *n*_12_=*k* heterozygotes, under Hardy-Weinberg equilibrium and fixed *n*_1_ and *n*_2_, is

$$\begin{array}{@{}rcl@{}} & \left(\frac{(2^{k+2})(n!)}{(\frac{n_{1}-k}{2}-1)!(k+2)!(\frac{n_{2}-k}{2}-1)!} \middle/ \frac{(2^{k})(n!)}{\frac{n_{1}-k}{2}!k!\frac{n_{2}-k}{2}!} \right)  \\ = & \frac{2^{k+2}}{2^{k}}\cdot \frac{n!}{n!}\cdot \frac{\frac{n_{1}-k}{2}!}{(\frac{n_{1}-k}{2}-1)!}\cdot \frac{k!}{(k+2)!}\cdot \frac{\frac{n_{2}-k}{2}!}{(\frac{n_{2}-k}{2}-1)!}  \\ = & 4\cdot 1\cdot \frac{n_{1}-k}{2}\cdot \frac{1}{(k+1)(k+2)}\cdot \frac{n_{2}-k}{2}  \\ = & \frac{(n_{1}-k)(n_{2}-k)}{(k+1)(k+2)}.  \end{array} $$

SNP-HWE also recognizes that it is unnecessary to start the computation with an accurate absolute likelihood for one table. Since the final p-value is computed as

$${\fontsize{8.5}{6}\begin{aligned} \frac{[\text{sum of null hypothesis likelihoods of at-least-as-extreme tables}]} {[\text{sum of null hypothesis likelihoods of all tables}]}, \end{aligned}} $$

it is fine for all computed likelihoods to be relative values off by a shared constant factor, since that constant factor will cancel out. This eliminates the need for log-gamma approximation.

**Figure 1:**
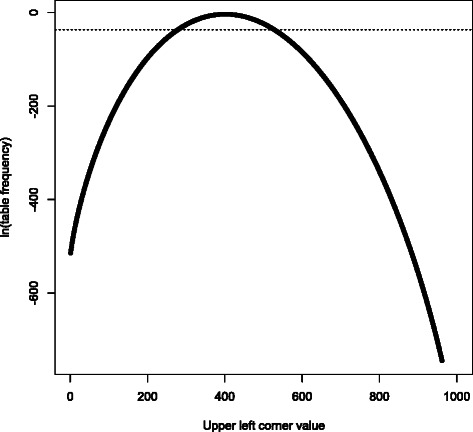
**2 × 2 contingency table log-frequencies.** This is a plot of relative frequencies of 2 × 2 contingency tables with top row sum 1000, left column sum 40000, and grand total 100000, reflecting a low-MAF variant where the difference between the chi-square test and Fisher’s exact test is relevant. All such tables with upper left value smaller than 278, or larger than 526, have frequency smaller than 2^−53^ (dotted horizontal line); thus, if the obvious summation algorithm is used, they have no impact on the p-value denominator due to numerical underflow. (It can be proven that this underflow has negligible impact on accuracy, due to how rapidly the frequencies decay.) A few more tables need to be considered when evaluating the numerator, but we can usually skip at least 70%, and this fraction improves as problem size increases.

While studying the software, we made two additional observations:

- Its size- *O*(*n*) memory allocation (where *n* is the sum of all contingency table entries) could be avoided by reordering the calculation; it is only necessary to track a few partial sums.
- Since likelihoods decay super-geometrically as one moves away from the most probable table, only $O(\sqrt {n})$ of the likelihoods can meaningfully impact the partial sums; the sum of the remaining terms is too small to consistently affect even the 10th significant digit in the final p-value. By terminating the calculation when all the partial sums stop changing (due to the newest term being too tiny to be tracked by IEEE-754 double-precision numbers), computational complexity is reduced from *O*(*n*) to $O(\sqrt {n})$ with no loss of precision. See Figure 1 for an example.

**Figure 2:**
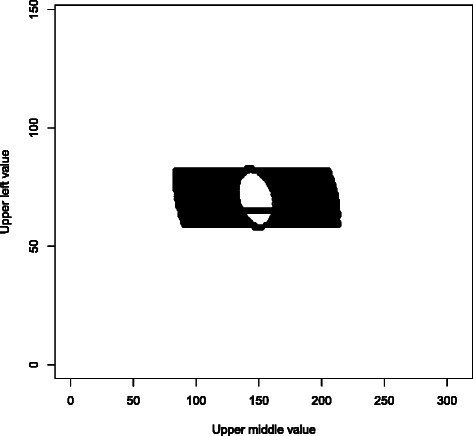
**Computation pattern for our 2 × 3 Fisher’s exact test implementation.** This is a plot of the set of alternative 2 × 3 contigency tables explicitly considered by our algorithm when testing the table with 65, 136, 324 in the top row and 81, 172, 314 in the bottom row. Letting *ℓ* denote the relative likelihood of observing the tested table under the null hypothesis, the set of tables with null hypothesis relative likelihoods between 2^−53^*ℓ* and *ℓ* has an ellipsoidal annulus shape, with area scaling as O(*n*) as the problem size increases; while the set of tables with relative likelihood greater than 2^−53^*l*_max_ (where *l*_max_ is the maximal single-table relative likelihood) has an elliptical shape, also with O(*n*) area. Summing the relative likelihoods in the first set, and then dividing that number by the sum of the relative likelihoods in the second set, yields the desired p-value to 10+ digit accuracy in O(*n*) time. In addition, we exploit the fact that a “row” of 2 × 3 table likelihoods sums to a single 2 × 2 table likelihood; this lets us essentially skip the top and bottom of the annulus, as well as all but a single row of the central ellipse.

PLINK 1.0 also has association analysis and quality control routines which perform Fisher’s exact test on 2×2 and 2×3 tables, using the FEXACT network algorithm from Mehta et al. [15,16]. The 2×2 case has the same mathematical structure as the Hardy-Weinberg equilibrium exact test, so it was straightforward to modify the early-termination SNP-HWE algorithm to handle it. The 2×3 case is more complicated, but retains the property that only $O(\sqrt {\mathrm {\# of tables}})$ relative likelihoods need to be evaluated, so we were able to develop a function to handle it in *O*(*n*) time; see Figure 2 for more details. Our timing data indicate that our new functions are consistently faster than both FEXACT and the update to the network algorithm by Requena et al. [17].

Standalone source code for early-termination SNP-HWE and Fisher’s 2×2/ 2×3 exact test is posted at [18]. Due to recent calls for use of mid-*p* adjustments in biostatistics [19,20], all of these functions have mid-*p* modes, and PLINK 1.9 exposes them.

We note that, while the Hardy-Weinberg equilibrium exact test is only of interest to geneticists, Fisher’s exact test has wider application. Thus, we are preparing another paper which discusses these algorithms in more detail, with proofs of numerical error bounds and a full explanation of how the Fisher’s exact test algorithm extends to larger tables.

##### Haplotype block estimation

It can be useful to divide the genome into blocks of variants which appear to be inherited together most of the time, since observed recombination patterns are substantially more “blocklike” than would be expected under a model of uniform recombination [21]. PLINK 1.0’s –blocks command implements a method of identifying these haplotype blocks by Gabriel et al. [22]. (More precisely, it is a restricted port of Haploview’s [23] implementation of the method).

This method is based on 90% confidence intervals (as defined by Wall and Pritchard [21]) for Lewontin’s *D*^′^ disequilibrium statistic for pairs of variants. Depending on the confidence interval’s boundaries, a pair of variants is classified as “strong linkage disequilibrium (LD)”, “strong evidence for historical recombination”, or “inconclusive”; then, contiguous groups of variants where “strong LD” pairs outnumber “recombination” pairs by more than 19 to 1 are greedily selected, starting with the longest base-pair spans.

**Figure 3:**
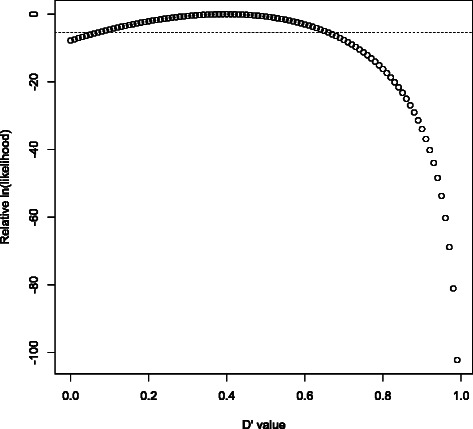
**Rapid classification of “recombination” variant pairs.** This is a plot of 101 equally spaced D’ log-likelihoods for (rs58108140, rs140337953) in 1000 Genomes phase 1, used in Gabriel et al.’s method of identifying haplotype blocks. Whenever the upper end of the 90% confidence interval is smaller than 0.90 (i.e. the rightmost 11 likelihoods sum to less than 5% of the total), we have strong evidence for historical recombination between the two variants. After determining that *L*(*D*^′^=*x*) has only one extreme value in [0, 1] and that it’s between 0.39 and 0.40, confirming *L*(*D*^′^=0.90)<*L*(*D*^′^=0.40)/220 is enough to finish classifying the variant pair (due to monotonicity: *L*(*D*^′^=0.90)≥*L*(*D*^′^=0.91)≥…≥*L*(*D*^′^=1.00)); evaluation of the other 99 likelihoods is now skipped in this case. The dotted horizontal line is at *L*(*D*^′^=0.40)/220.
- Instead of saving the classification of every variant pair and looking up the resulting massive table at a later point, we just update a small number of “strong LD pairs within last *k* variants” and “recombination pairs within last *k* variants” counts while processing the data sequentially, saving only final haploblock candidates. This reduces the amount of time spent looking up out-of-cache memory, and also allows much larger datasets to be processed.
- Since “strong LD” pairs must outnumber “recombination” pairs by 19 to 1, it does not take many “recombination” pairs in a window before one can prove no haploblock can contain that window. When this bound is crossed, we take the opportunity to entirely skip classification of many pairs of variants.

PLINK 1.9 accelerates this in several ways:

- Estimation of diplotype frequencies and maximum-likelihood *D*^′^ has been streamlined. Bit population counts are used to fill the contingency table; then we use the analytic solution to Hill’s diplotype frequency cubic equation [24,25] and only compute and compare log likelihoods in this step when multiple solutions to the equation are in the valid range.
- 90% confidence intervals were originally estimated by computing relative likelihoods at 101 points (corresponding to *D*^′^=0,*D*^′^=0.01,…,*D*^′^=1) and checking where the resulting cumulative distribution function (cdf) crossed 5% and 95%. However, the likelihood function rarely has more than one extreme point in (0,1) (and the full solution to the cubic equation reveals the presence of additional extrema); it is usually possible to exploit this unimodality to establish good bounds on key cdf values after evaluating just a few likelihoods. In particular, many confidence intervals can be classified as “recombination” after inspection of just two of the 101 points; see Figure 3.

Most of these ideas are implemented in haploview_blocks_classify() and haploview_blocks() in plink_ld.c. The last two optimizations were previously implemented in Taliun’s “LDExplorer” R package [26].

##### Coordinate-descent LASSO

PLINK 1.9 includes a basic coordinate-descent LASSO implementation [27] (–lasso), which can be useful for phenotypic prediction and related applications. See Vattikuti et al. for discussion of its theoretical properties [28].

#### Newly integrated third-party software

##### PLINK 1.0 commands

Many teams have significantly improved upon PLINK 1.0’s implementations of various commands and made their work open source. In several cases, their innovations have been integrated into PLINK 1.9; examples include

- Pahl et al.’s PERMORY algorithm for fast permutation testing [29],
- Wan et al.’s BOOST software for fast epistasis testing [30],
- Ueki, Cordell, and Howey’s –fast-epistasis variance correction and joint-effects test [31,32],
- Taliun, Gamper, and Pattaro’s optimizations to Gabriel et al.’s haplotype block identification algorithm (discussed above) [26], and
- Pascal Pons’s winning submission to the GWAS Speedup logistic regression crowdsourcing contest [33]. (The contest was designed by Po-Ru Loh, run by Babbage Analytics & Innovation and TopCoder, and subsequent analysis and code preparation were performed by Andrew Hill, Ragu Bharadwaj, and Scott Jelinsky. A manuscript is in preparation by these authors and Iain Kilty, Kevin Boudreau, Karim Lakhani and Eva Guinan.)

In all such cases, PLINK’s citation instructions direct users of the affected functions to cite the original work.

##### Multithreaded gzip

For many purposes, compressed text files strike a good balance between ease of interpretation, loading speed, and resource consumption. However, the computational cost of generating them is fairly high; it is not uncommon for data compression to take longer than all other operations combined. To make a dent in this bottleneck, we have written a simple multithreaded compression library function based on Mark Adler’s excellent pigz program [34], and routed most of PLINK 1.9’s gzipping through it. See parallel_compress() in pigz.c for details.

#### Convenience features

##### Import and export of Variant Call Format (VCF) and Oxford-formatted data

PLINK 1.9 can import data from Variant Call Format (–vcf), binary VCF (–bcf), and Oxford-format (–data, –bgen) files. However, since it cannot handle genotype likelihoods, phase information or variants with more than two alleles, the import process can be quite lossy. Specifically,

- With Oxford-format files, genotype likelihoods smaller than 0.9 are normally treated as missing calls, and the rest are treated as hard calls. –hard-call-threshold can be used to change the threshold, or request independent pseudorandom calls based on the likelihoods in the file.
- Phase is discarded.
- By default, when a VCF variant has more than one alternate allele, only the most common alternate is retained; all other alternate calls are converted to missing. –biallelic-only can be used to skip variants with multiple alternate alleles.

Export to these formats is also possible, via –recode vcf and –recode oxford.

##### Unplaced contig and nonhuman species support

When the –allow-extra-chr or –aec flag is used, PLINK 1.9 allows datasets to contain unplaced contigs or other arbitrary chromosome names, and most commands will handle them in a reasonable manner. Also, arbitrary nonhuman species (with haploid or diploid genomes) can now be specified with –chr-set.

##### Command-line help

To improve the experience of using PLINK interactively, we have expanded the –help flag’s functionality. When invoked with no parameters, it now prints an entire mini-manual. Given keyword(s), it instead searches for and prints mini-manual entries associated with those keyword(s), and handles misspelled keywords and keyword prefixes in a reasonable manner.

### A comment on within-family analysis

Most of our discussion has addressed computational issues. However, there is one methodological issue that deserves a brief comment. The online documentation of PLINK 1.07 weighed the pros and cons of its permutation procedure for within-family analysis of quantitative traits (QFAM) with respect to the standard quantitative transmission disequilibrium test (QTDT) [35]. It pointed out that likelihood-based QTDT enjoyed the advantages of computational speed and increased statistical power. However, a comparison of statistical power is only meaningful if both procedures are anchored to the same Type 1 error rate with respect to the null hypothesis of no linkage with a causal variant, and Ewens et al. has shown that the QTDT is not robust against certain forms of confounding (population stratification) [36]. On the other hand, the validity of a permutation procedure such as QFAM only depends on the applicability of Mendel’s laws. When this nicety is combined with the vast speedup of permutation in PLINK 1.9, a given user may now decide to rate QFAM more highly relative to QTDT when considering available options for within-family analysis.

### Performance comparisons

In the following tables, running times are collected from seven machines operating on three datasets.

- “Mac-2” denotes a MacBook Pro with a 2.8 Ghz Intel Core 2 Duo processor and 4GB RAM running OS X 10.6.8.
- “Mac-12” denotes a Mac Pro with two 2.93 Ghz Intel 6-core Xeon processors and 64GB RAM running OS X 10.6.8.
- “Linux32-2” denotes a machine with a 2.4 Ghz Intel Core 2 Duo E6600 processor and 1GB RAM running 32-bit Ubuntu Linux.
- “Linux32-8” denotes a machine with a 3.4 Ghz Intel Core i7-3770 processor (8 cores) and 8GB RAM running 32-bit Ubuntu Linux.
- “Linux64-512” denotes a machine with sixty-four AMD 8-core Opteron 6282 SE processors and 512GB RAM running 64-bit Linux.
- “Win32-2” denotes a laptop with a 2.4 Ghz Intel Core i5-2430 M processor (2 cores) and 4GB RAM running 32-bit Windows 7 SP1.
- “Win64-2” denotes a machine with a 2.3 Ghz Intel Celeron G1610T processor (2 cores) and 8GB RAM running 64-bit Windows 8.
- “synth1” refers to a 1000 sample, 100000 variant synthetic dataset generated with HAPGEN2 [37], while “synth1p” refers to the same dataset after one round of –indep-pairwise 50 5 0.5 pruning (with 76124 markers remaining). For case/control tests, PLINK 1.9’s –tail-pheno 0 command was used to downcode the quantitative phenotype to case/control.
- “synth2” refers to a 4000 case, 6000 control synthetic dataset with 88025 markers on chromosomes 19-22 generated by resampling HapMap and 1000 Genomes data with simuRare [38] and then removing monomorphic loci. “synth2p” refers to the same dataset after one round of –indep-pairwise 700 70 0.7 pruning (with 71307 markers remaining).
- “1000g” refers to the entire 1092 sample, 39637448 variant 1000 Genomes project phase 1 dataset [39]. “chr1” refers to chromosome 1 from this dataset, with 3001739 variants. “chr1snp” refers to chromosome 1 after removal of all non-SNPs and one round of –indep-pairwise 20000 2000 0.5 pruning (798703 markers remaining). Pedigree information was not added to these datasets before our tests.

All times are in seconds. To reduce disk-caching variance, timing runs are preceded by “warmup” commands like plink –freq. PLINK 1.07 was run with the –noweb flag. “nomem” indicates that the program ran out of memory and there was no low-memory mode or other straightforward workaround. A tilde indicates that runtime was extrapolated from several smaller problem instances.

#### Initialization and basic I/O

Table 1 displays execution times for plink –freq, one of the simplest operations PLINK can perform. These timings reflect fixed initialization and I/O overhead. (Due to the use of warmup runs, they do not include disk latency).

**Table 1:** **–freq** **times (sec)**

Dataset	Machine	PLINK 1.07	PLINK 1.90	Ratio
synth1	Mac-2	7.3	0.24	30
	Mac-12	6.2	0.18	34
	Linux32-2	13.1	0.56	23
	Linux32-8	4.3	0.18	24
	Linux64-512	5.4	0.18	27
	Win32-2	14.3	0.68	21
	Win64-2	9.6	0.33	29
synth2	Mac-2	43.3	0.84	52
	Mac-12	38.2	0.34	110
	Linux32-2	80.1	1.9	42
	Linux32-8	25.2	0.53	48
	Linux64-512	34.1	0.40	85
	Win32-2	83.6	1.3	64
	Win64-2	70.8	0.55	130
chr1snp	Mac-2	52.5	3.5	15
	Mac-12	40.5	1.3	31
	Linux32-2	72.9	10.2	7.15
	Linux32-8	29.7	1.4	21
	Linux64-512	36.8	1.4	26
	Win32-2	104.3	4.5	23
	Win64-2	76.8	2.2	35
chr1	Mac-2	403.9	35.0	11.5
	Mac-12	163.9	5.3	31
	Linux32-2	nomem	65.3	
	Linux32-8	134.1	12.8	10.5
	Linux64-512	144.7	5.4	27
	Win32-2	389.2	21.4	18.2
	Win64-2	285.3	8.1	35

This command reports allele frequencies for each variant. The computation is trivial, so the timings just reflect program initialization speed and file I/O efficiency.

#### Identity-by-state matrices, complete linkage clustering

The PLINK 1.0 –cluster –matrix flag combination launches an identity-by-state matrix calculation and writes the result to disk, and then performs complete linkage clustering on the data; when –ppc is added, a pairwise population concordance constraint is applied to the clustering process. As discussed earlier, PLINK 1.9 employs an XOR/bit population count algorithm which speeds up the matrix calculation by a large constant factor; the computational complexity of the clustering algorithm has also been reduced, from *O*(*n*^3^) to *O*(*n*^2^ log*n*). (Further improvement of clustering complexity, to *O*(*n*^2^), is possible in some cases [40].)

In Table 2, we compare PLINK 1.07 and PLINK 1.9 execution times under three scenarios: identity-by-state (IBS) matrix calculation only (–cluster –matrix –K [sample count - 1] in PLINK 1.07, –distance ibs square in PLINK 1.9), IBS matrix + standard clustering (–cluster –matrix for both versions), and identity-by-descent (IBD) report generation (–Z-genome.)

**Table 2:** **Identity-by-state (Hamming distance) and complete linkage clustering times (sec)**

Calculation	Dataset	Machine	PLINK 1.07	PLINK 1.90	Ratio
IBS matrix only	synth1p	Mac-2	2233.6	1.9	1.2 k
		Mac-12	1320.4	1.2	1.1 k
		Linux32-8	1937.2	2.8	690
		Linux64-512	1492	3.7	400
		Win32-2	3219.0	7.2	450
		Win64-2	2674.4	1.5	1.8 k
	synth2p	Mac-2	∼190 k	118.8	1.6 k
		Mac-12	∼99 k	23.5	4.2 k
		Linux32-8	152.5 k	214.3	710
		Linux64-512	∼98 k	25.3	3.9 k
		Win32-2	∼270 k	654.5	410
		Win64-2	∼200 k	104.6	1.9 k
	chr1snp	Mac-2	∼26 k	17.5	1.5 k
		Mac-12	13.4 k	12.6	1.06 k
		Linux32-8	18.4 k	30.9	600
		Linux64-512	∼14 k	43.1	320
		Win32-2	32.7 k	95.9	341
		Win64-2	∼26 k	15.3	1.7 k
Basic clustering	synth1p	Mac-2	2315.7	2.7	860
		Mac-12	1317.9	2.0	660
		Linux32-8	1898.7	4.1	460
		Linux64-512	1496	4.5	330
		Win32-2	3301.7	9.1	360
		Win64-2	2724.5	1.9	1.4 k
	synth2p	Mac-2	∼230 k	245.6	940
		Mac-12	∼140 k	123.9	1.1 k
		Linux32-8	197.1 k	395.6	498
		Linux64-512	∼125 k	143.3	872
		Win32-2	∼440 k	976.7	450
		Win64-2	∼270 k	127.9	2.1 k
	chr1snp	Mac-2	∼26 k	18.4	1.4 k
		Mac-12	13.6 k	13.5	1.01 k
		Linux32-8	18.5 k	33.4	554
		Linux64-512	∼14 k	44.2	320
		Win32-2	33.2 k	95.0	349
		Win64-2	∼26 k	15.8	1.6 k
IBD report	synth1p	Mac-2	2230.1	12.4	180
		Mac-12	1346.2	2.4	560
		Linux32-8	2019.9	12.4	163
		Linux64-512	1494	5.0	300
		Win32-2	3446.3	42.2	81.7
		Win64-2	2669.8	15.1	177
	synth2p	Mac-2	∼190 k	447.1	420
		Mac-12	∼99 k	50.3	2.0 k
		Linux32-8	161.4 k	618.7	261
		Linux64-512	∼98 k	57.4	1.7 k
		Win32-2	∼270 k	1801.1	150
		Win64-2	∼200 k	541.0	370
IBD report	chr1snp	Mac-2	∼26 k	24.8	1.0 k
		Mac-12	13.4 k	14.6	918
		Linux32-8	18.5 k	53.5	346
		Linux64-512	∼14 k	46.5	300
		Win32-2	33.1 k	199.2	166
		Win64-2	∼26 k	25.1	1.0 k

Computation of the basic distance matrix is expensive, but has an “embarrassingly parallel” structure. Clustering requires an additional serial step, while the identity-by-descent report includes a pairwise population concordance test which does not benefit from bit-level parallelism, but speedups for both remain greater than 100x on 64-bit systems.

(Note that newer algorithms such as BEAGLE’s fastIBD [41] generate more accurate IBD estimates than PLINK –Z-genome. However, the –Z-genome report contains other useful information.)

#### Genomic relationship matrices

GCTA’s –make-grm-bin command (–make-grm in early versions) calculates the variance-standardized genomic relationship matrix used by many of its other commands. The latest implementation as of this writing (v1.24) is very fast, but only runs on 64-bit Linux, uses single- instead of double-precision arithmetic, and has a high memory requirement.

PLINK 1.9’s implementation of this calculation is designed to compensate for GCTA 1.24’s limitations—it is cross-platform, works in low-memory environments, and uses double-precision arithmetic while remaining within a factor of 2-5 on speed. See Table 3 for timing data. The comparison is with GCTA 1.24 on 64-bit Linux, and v1.02 elsewhere.

**Table 3:** **Genomic relationship matrix calculation times (sec)**

Dataset	Machine	GCTA	PLINK 1.90	Ratio
synth1p	Mac-2	222.2	7.2	31
	Mac-12	184.7	5.0	37
	Linux32-8	248.4	10.9	22.8
	Linux64-512	4.4	9.6	0.46
	Win32-2	373.1	39.3	9.5
	Win64-2	367.2	6.6	56
synth2p	Mac-2	nomem	805.8	
	Mac-12	17.0 k	138.3	123
	Linux32-8	nomem	1153.4	
	Linux64-512	65.1	318.9	0.20
	Win32-2	nomem	2007.2	
	Win64-2	nomem	450.1	
chr1snp	Mac-2	nomem	87.1	
	Mac-12	2260.9	50.9	44.4
	Linux32-8	nomem	94.3	
	Linux64-512	58.3	91.6	0.64
	Win32-2	nomem	317.5	
	Win64-2	nomem	65.7	

This involves a variance-normalizing distance function which cannot be efficiently computed with just bit population counts. PLINK 1.9’s lookup table-based algorithm is slower than GCTA 1.24 on heavily multicore machines (see the Linux64-512 results), but has complementary advantages in portability, accuracy, and memory efficiency.

#### Linkage disequilibrium-based variant pruning

The PLINK 1.0 –indep-pairwise command is frequently used in preparation for analyses which assume approximate linkage equilibrium. In Table 4, we compare PLINK 1.07 and PLINK 1.9 execution times for some reasonable parameter choices. The *r*^2^ threshold for “synth2” was chosen to make the “synth1p” and “synth2p” pruned datasets contain similar number of SNPs, so Tables 2 and 3 could clearly demonstrate scaling with respect to sample size.

**Table 4:** **–indep-pairwise** **runtimes (sec)**

Parameters	Dataset	Machine	PLINK 1.07	PLINK 1.90	Ratio
50 5 0.5	synth1	Mac-2	701.3	0.63	1.1 k
		Mac-12	569.4	0.55	1.0 k
		Linux32-8	572.7	0.95	600
		Linux64-512	462	0.60	770
		Win32-2	1163.9	3.2	360
		Win64-2	1091.9	1.0	1.1 k
700 70 0.7	synth2	Mac-2	∼120 k	31.9	3.8 k
		Mac-12	63.0 k	20.6	3.06 k
		Linux32-8	57.4 k	66.0	870
		Linux64-512	∼120 k	26.4	4.5 k
		Win32-2	139.3 k	127.3	1.09 k
		Win64-2	∼200 k	22.9	8.7 k
20000 2000 0.5	chr1	Mac-2	nomem	1520.1	
		Mac-12	nomem	1121.7	
		Linux32-8	nomem	4273.9	
		Linux64-512	∼950 k	1553.3	610
		Win32-2	nomem	4912.7	
		Win64-2	nomem	1205.1	
	1000g	Mac-2	nomem	20.5 k	
		Mac-12	nomem	14.5 k	
		Linux32-8	nomem	54.5 k	
		Linux64-512	∼13000 k	20.2 k	640
		Win32-2	nomem	64.5 k	
		Win64-2	nomem	14.7 k	

This command is used to select a set of genetic markers which are not too highly correlated with one another. The PLINK 1.9 implementation benefits from laziness (i.e. the correlation coefficient between a pair of variants is no longer computed when it is not needed by the main pruning algorithm) and bitwise operations.

#### Haplotype block estimation

Table 5 demonstrates the impact of our rewrite of –blocks. Due to a minor bug in PLINK 1.0’s handling of low-MAF variants, we pruned each dataset to contain only variants with MAF ≥0.05 before running –blocks. 95506 markers remained in the “synth1” dataset, and 554549 markers remained in “chr1”. A question mark indicates that the extrapolated runtime may not be valid since we suspect Haploview or PLINK 1.07 would have run out of memory before finishing.

**Table 5:** **–blocks** **runtimes (sec)**

Parameters	Dataset	Machine	Haploview 4.2	PLINK 1.07	PLINK 1.90
–ld-window-kb 500	synth1	Mac-2	nomem	3198.4	1.7
		Mac-12	∼45 k	3873.0	1.3
		Linux32-2	nomem	5441.1	3.4
		Linux64-512	∼57 k	2323.4	2.9
		Win32-2	nomem	9803.4	8.9
		Win64-2	∼51 k	5513.4	2.8
–ld-window-kb 1000	synth1	Mac-2	nomem	6185.7	2.2
		Mac-12	∼45 k	7394.4	9.8
		Linux32-2	nomem	9876.8	10.0
		Linux64-512	∼57 k	4462.1	3.9
		Win32-2	nomem	18925.7	17.3
		Win64-2	∼51 k	10.3 k	3.6
–ld-window-kb 500	chr1	Mac-2	nomem	∼2700 k?	550.9
		Mac-12	nomem	∼3600 k?	426.0
		Linux32-2	nomem	∼4300 k?	1288.4
		Linux64-512	∼440 k?	∼2600 k?	1119.7
		Win32-2	nomem	∼17000 k?	4535.8
		Win64-2	nomem	∼5700 k?	1037.2

This command breaks the genome into estimated “haplotype blocks” which are usually inherited together. The PLINK 1.9 implementation combines optimizations recently developed by Taliun et al. [26] with additional laziness and bit-level parallelism.

#### Association analysis max(T) permutation tests

PLINK 1.0’s basic association analysis commands were quite flexible, but the powerful max(T) permutation test suffered from poor performance. PRESTO [42] and PERMORY introduced major algorithmic improvements (including bit population count) which largely solved the problem. Table 6 demonstrates that PLINK 1.9 successfully extends the PERMORY algorithm to the full range of PLINK 1.0’s association analyses, while making Fisher’s exact test practical to use in permutation tests. (There is no 64-bit Windows PERMORY build, so the comparisons on the Win64-2 machine are between 64-bit PLINK and 32-bit PERMORY.)

**Table 6:** **Association analysis max(T) permutation test times (sec)**

Other parameter(s)	Dataset	Machine	PLINK 1.07	PERMORY 1.1	PLINK 1.90	Ratio
–trend (C/C)	synth1	Mac-2	∼20 k		18.7	1.1 k
		Mac-12	∼16 k		2.8	5.7 k
		Linux32-2	∼21 k		65.0	320
		Linux64-512	∼17 k	285.0	2.8	
		Win32-2	∼35 k	1444.2	61.5	
		Win64-2	∼25 k	889.7	14.4	
	synth2	Mac-2	∼170 k		42.4	4.0 k
		Mac-12	∼180 k		6.4	28 k
		Linux32-2	∼410 k		391.0	1.0 k
		Linux64-512	∼200 k	580.9	13.7	
		Win32-2	∼1100 k	2362.5	198.0	
		Win64-2	∼370 k	1423.6	34.0	
–fisher (C/C)	synth1	Mac-2	∼150 k		21.9	6.9 k
		Mac-12	∼150 k		3.7	41 k
		Linux32-2	∼170 k		57.8	2.9 k
		Linux64-512	∼120 k		3.4	35 k
		Win32-2	∼440 k		64.9	6.8 k
		Win64-2	∼200 k		22.0	9.1 k
	synth2	Mac-2	∼890 k		49.8	18 k
		Mac-12	∼690 k		7.6	91 k
		Linux32-2	∼1300 k		393.7	3.3 k
		Linux64-512	∼720 k		13.0	55 k
		Win32-2	∼3600 k		208.3	17 k
		Win64-2	∼1700 k		35.6	48 k
–assoc (QT)	synth1	Mac-2	∼30 k		148.0	200
		Mac-12	∼22 k		22.6	970
		Linux32-2	∼68 k		847.2	80
		Linux64-512	∼29 k		29.2	990
		Win32-2	∼58 k		896.1	65
		Win64-2	∼36 k		264.2	140
–assoc lin (QT)	synth1	Mac-2			606.8	
		Mac-12			34.7	
		Linux32-2			3212.6	
		Linux64-512		1259.8	46.4	27.2
		Win32-2		2115.7	3062.7	0.69
		Win64-2		972.6	336.6	2.89

All runs are with 10000 permutations and –seed 1. The PLINK 1.9 implementation extends Pahl et al.’s PERMORY algorithm [29] with multithreading (note the 12- and 512-core machine results) and additional low-level optimizations.

### PLINK 2.0 design

Despite its computational advances, we recognize that PLINK 1.9 can ultimately still be an unsatisfactory tool for working with imputed genomic data, due to the limitations of the PLINK 1 binary file format. To address this, we designed a new core file format capable of representing most of the information emitted by modern imputation tools, which is the cornerstone of our plans for PLINK 2.0.

#### Multiple data representations

As discussed earlier, PLINK 1 binary is inadequate in three ways: likelihoods strictly between 0 and 1 cannot be represented, phase information cannot be stored, and variants are limited to two alleles. This can be addressed by representing *all* calls probabilistically, and introducing a few other extensions. Unfortunately, this would make PLINK 2.0’s representation of PLINK 1-format data so inefficient that it would amount to a serious downgrade from PLINK 1.9 for many purposes.

Therefore, our new format defines several data representations, one of which is equivalent to PLINK 1 binary, and allows different files, or even variants within a single file, to use different representations. To work with this, PLINK 2.0 will include a translation layer which allows individual functions to assume a specific representation is used. As with the rest of PLINK’s source code, this translation layer will be GPLv3-licensed open source; and unlike most of the other source code, we are explicitly designing it to be usable as a standalone library. PLINK 2.0 will also be able to convert files/variants from one data representation to another, making it practical for third-party tools lacking access to the library to demand a specific representation.

#### Reference vs. alternate alleles

The now-ubiquitous VCF file format requires reference alleles to be distinguished from alternate alleles, and an increasing number of software tools and pipelines do not tolerate scrambling of the two. This presents an interoperability problem for PLINK: while it was theoretically possible to handle binary data with PLINK 1.0 in a manner that preserved the reference vs. alternate allele distinction when it was originally present, with constant use of –keep-allele-order and related flags, doing so was inconvenient and error-prone, especially since the accompanying native.ped/.map and.tped/.tfam text formats had no place to store that information. PLINK 1.9’s –a2-allele flag, which can import that information from a VCF file, provides limited relief, but it is still necessary for users to fight against the program’s major/minor-allele based design.

We aim to solve this problem for good in PLINK 2.0. The file format explicitly defines reference vs. alternate alleles, and this information will be preserved across runs by default. In addition, the file format will include a flag distinguishing provisional reference allele assignments from those derived from an actual reference genome. When PLINK 2.0 operates on.ped/.map or similar data lacking a reference vs. alternate distinction, it will treat a highest-frequency allele as the reference, while flagging it as a provisional assignment. When a file with flagged-as-provisional reference alleles is merged with another file with unflagged reference alleles, the unflagged reference allele assignments take precedence. (Merges involving conflicting unflagged reference alleles will fail unless the user specifies which source file takes precedence.) It will also be straightforward to import real reference allele assignments with an analogue of –a2-allele.

#### Data compression

PLINK 1.9 demonstrates the power of a weak form of compressive genomics [43]: by using bit arithmetic to perform computation directly on compressed genomic data, it frequently exhibits far better performance than programs which require an explicit decompression step. But its “compressed format” is merely a tight packing which does not support the holy grail of true sub-linear analysis.

To do our part to make “strong” sub-linear compressive genomics a reality, the PLINK 2 file format will introduce support for “deviations from most common value” storage of low-MAF variants. For datasets containing many samples, this captures much of the storage efficiency benefit of having real reference genomes available, without the drawback of forcing all programs operating on the data to have access to a library of references. Thanks to PLINK 2.0’s translation layer and file conversion facilities, programmers will be able to ignore this feature during initial development of a tool, and then work to exploit it after basic functionality is in place.

We note that LD-based compression of variant groups is also possible, and Sambo’s SNPack software [44] applies this to the PLINK 1 binary format. We do not plan to support this in PLINK 2.0 due to the additional software complexity required to handle probabilistic and multiallelic data, but we believe this is a promising avenue for development and look forward to integrating it in the future.

#### Remaining limitations

PLINK 2.0 is designed to meet the needs of tomorrow’s genome-wide association studies and population-genetics research; in both contexts, it is appropriate to apply a single genomic coordinate system across all samples, and preferred sample sizes are large enough to make computational efficiency a serious issue.

Whole-exome and whole-genome sequencing also enables detailed study of structural variations which defy clean representation under a single coordinate system; and the number of individuals in such studies is typically much smaller than the tens or even hundreds of thousands which are sometimes required for effective GWAS. There are no plans to make PLINK suitable for this type of analysis; we strongly recommend the use of another software package, such as PLINK/SEQ [45], which is explicitly designed for it. This is why the PLINK 2 file format will still be substantially less expressive than VCF.

An important consequence is that, despite its ability to import and export VCF files, PLINK should not be used for management of genomic data which will be subject to both types of analysis, because it discards all information which is not relevant for its preferred type. However, we will continue to extend PLINK’s ability to interpret VCF-like formats and interoperate with other popular software.

## Availability and requirements

- **Project name:** Second-generation PLINK
- **Project (source code) home page:**https://www.cog-genomics.org/plink2/(https://github.com/chrchang/plink-ng)
- **Operating systems:** Linux (32/64-bit), OS X (64-bit Intel), Windows (32/64-bit)
- **Programming language:** C, C++
- **Other requirements (when recompiling):** GCC version 4, a few functions also require LAPACK 3.2
- **License:** GNU General Public License version 3.0 (GPLv3)
- **Any restrictions to use by non-academics:** none

## Availability of supporting data

The test data and the source code snapshots supporting the results of this article are available in the *GigaScience* repository, GigaDB [8].

## Additional file

Additional file 1
**Detailed description of software bit population count, as applied to identity-by-state computation.**

## Abbreviations

PLINK: The software toolset that is the main subject of this paper. The name was originally shorthand for “population linkage”
BEAGLE: A software package capable of high-accuracy haplotype phasing, genotype imputation, and identity-by-descent estimation, developed by Browning [2]
GCTA: Genome-wide Complex Trait Analysis. This refers to both the statistical method and the software implementation discussed in [7]
VCF: Variant Call Format [5]
x86: A family of backward compatible instruction set architectures based on the Intel 8086 CPU
IBS: Identity-by-state. A simple measure of genomic similarity, equal to the number of identical alleles divided by the number of observations
popcount: Bit population count. The number of ’1’ bits in a bit vector
XOR: Exclusive-or. A binary logical operation that evaluates to true if exactly one of its arguments is true
SSE2: Streaming SIMD Extensions 2. A SIMD (single instruction, multiple data) processor supplementary instruction set first introduced by Intel with the initial version of the Pentium 4 in 2001
GPU: Graphics processing unit
HWE: Hardy-Weinberg equilibrium
SNP: Single-nucleotide polymorphism
FEXACT: A network algorithm for evaluating Fisher’s exact test p-values, developed by Mehta et al. [15,16]
LD: Linkage disequilibrium
PERMORY: A software package designed to perform efficient permutation tests for large-scale genetic data sets, developed by Pahl et al. [29]
GWAS: Genome-Wide Association Study
QFAM: A family-based quantitative trait association analysis procedure, introduced by PLINK 1.0, which combines a simple linear regression of phenotype on genotype with a special permutation test which corrects for family structure
QTDT: Quantitative Transmission Disequilibrium Tests, developed primarily by Abecasis et al. [35]
Ghz: Gigahertz
GB: Gigabyte
RAM: Random-access memory
I/O: Input/output
MAF: Minor allele frequency. Frequency of the least common allele that is still present in a population
GPLv3: GNU General Public License version 3
