# Supplementary material for: Second-generation PLINK: rising to the challenge of larger and richer datasets
Source: Gigascience. 2015 Feb 25;4:7. doi: 10.1186/s13742-015-0047-8 (PMC4342193; doi:10.1186/s13742-015-0047-8)
Supplement: Additional file 1 — Detailed description of software bit population count, as applied to identity-by-state computation. [file 13742_2015_47_MOESM1_ESM.pdf]

Identity-by-state and software popcount

PLINK 1.9's most important optimization is its replacement of slow loops iterating over single genotype calls with bitwise operations on many calls at a time. This document illustrates how identity-by-state (i.e. Hamming distance) between two genomes is computed in this fashion.

Step 1: Transposition and other preprocessing. PLINK's core file format saves genotype calls in a variant-major manner. IBS computation is faster with sample-major data, and its overall time complexity is $O(mn^2)$ while transposition is just $O(mn)$, so we transpose the data before the main loop. We also assemble a bit array tracking the presence of missing genotype calls.

Vincent:

`11|11|10|10|01|11|11|00|11|11|00|11|11|11|10|11`

`genotypes`

`11|11|11|11|00|11|11|11|11|11|11|11|11|11|11|11`

`nonmissingness`

Anton:

`01|11|11|11|01|11|11|11|11|11|10|11|11|11|11|11`

`genotypes`

`00|11|11|11|00|11|11|11|11|11|11|11|11|11|11|11`

`nonmissingness`

Step 2: XOR-and-mask. PLINK 1 represents homozygous major calls with binary `11`, heterozygous calls with `10`, and homozygous minor calls with `00`. Conveniently, if you take the exclusive-or of two such values, the number of set bits in the result is the number of differing allele calls; thus, the overall Hamming distance between two genomes in (transposed) PLINK 1 format is the bit population count of their XOR. Excepting missing calls (represented by `01`), that is; we "mask" (via AND operations) the final result with both nonmissingness arrays to force those bits to zero. (The red '0' below is due to the mask.)

`0|0|0|0|0|1|0|1|0|0|0|0|0|0|1|1|0|0|0|0|1|0|0|0|0|0|0|0|0|1|0|0`

`XOR-and-mask result`

Step 3: Software popcount. Since PLINK is still used on many machines lacking a hardware popcount instruction, we use SSE2 (in x86-64 builds) or basic word (in 32-bit builds) operations to implement the "bitslice" algorithm discussed by Dalke et al., which is almost as fast when acting on long arrays. For clarity of exposition, we illustrate what happens with six 32-bit words; our SSE2 code applies the same idea to batches of fifteen or thirty 128-bit blocks.

```
  00000101000000110000100000000100
                                W1,1
  11001010100000101000101100100100
                                W1,2
  10001100010000110001001001110000
                                W1,3
  00000000000000000000001000000010
                                W1,4
  00000100000001001111000000100100
                                W1,5
  01110001001001010000100100010010
                                W1,6
```

This can be seen as a collection of 192 one-bit values which add up to our desired result.

The bitslice algorithm starts by generating a collection of two-bit partial sums which add up to the same total. Specifically, the partial sums in $W_{2,1}$ aggregate two bits in $W_{1,1}$ and an even-position bit in $W_{1,3}$; $W_{2,2}$ aggregates pairs of bits in $W_{1,2}$ and odd-position bits in $W_{1,3}$; $W_{2,3}$ aggregates pairs of bits in $W_{1,4}$ and even-position bits in $W_{1,6}$; and $W_{2,4}$ aggregates pairs of bits in $W_{1,5}$ and odd-position bits in $W_{1,6}$. The actual operations are a right-shift-1, a mask with `010101`..., a subtraction, a mask (even-position) or right-shift-1-and-mask (odd-position) with `010101`..., and an addition.

```
  00000101000000110000100000000100
                                W1,1
- 00000000000000010000010000000000
          W1,1 after right-shift-1 and 010101.... mask
=  0 0 1 1 0 0 0 2 0 0 1 0 0 0 1 0

+ 00000100010000010001000001010000
                   W1,3 after 010101... mask
=  0 0 2 1 1 0 0 3 0 1 1 0 1 1 1 0
                                W2,1
```

Note that "2" is shorthand for binary `10` and "3" is shorthand for binary `11` here; similar shorthand will be used for four- and eight-bit partial sums on the next page.

The next step is to use these to produce an even smaller collection of four-bit partial sums with the same total. Specifically, $W_{4,1}$ aggregates two values in $W_{2,1}$ and two values in $W_{2,2}$, while $W_{4,2}$ aggregates two values in $W_{2,3}$ and two values in $W_{2,4}$.

	0	0	0	1	0	0	0	3	0	1	0	0	0	1	0	0	$W_{2,1}$ after 001100110011... mask
+	0	0	0	2	0	1	0	0	0	0	0	1	0	1	0	1	$W_{2,1}$ after right-shift-2 and 001100110011... mask
+	0	0	0	1	0	0	0	2	0	0	0	3	0	2	0	0	$W_{2,2}$ after 001100110011... mask
+	0	3	0	2	0	1	0	0	0	1	0	1	0	0	0	1	$W_{2,2}$ after right-shift-2 and 001100110011... mask
=		3		6		2		5		2		5		4		2	$W_{4,1}$

Then we produce a single word of eight-bit partial sums from $W_{4,1}$ and $W_{4,2}$. (Since none of the four-bit partial sums can be greater than 12, and eight bits can represent values up to 255, we can actually merge up to 10 pairs of partial sums at this stage, rather than just 2; this is done by some of our SSE2 code.)

	0	6	0	5	0	5	0	2	$W_{4,1}$ after 00001111000011110000111100001111 mask
+	0	3	0	2	0	2	0	4	$W_{4,1}$ after right-shift-4 and mask
+	0	2	0	3	0	3	0	3	$W_{4,2}$ after mask
+	0	3	0	1	0	4	0	2	$W_{4,2}$ after right-shift-4 and mask
=		14		11		14		11	W_8

Finally, we add these eight-bit partial sums: 14 + 11 + 14 + 11 = 50, which is indeed the number of set bits among the original 192.
